# A Mathematical Model for Suppression Subtractive Hybridization

**DOI:** 10.1002/cfg.206

**Published:** 2002-10

**Authors:** Chetan Gadgil, Anette Rink, Craig Beattie, Wei-Shou Hu

**Affiliations:** 1 Department of Chemical Engineering and Materials Science 421 Washington Avenue SE University of Minnesota Minneapolis MN 55455 USA; 2 Department of Animal Biotechnology School of Veterinary Medicine University of Nevada Reno NV 89557 USA

## Abstract

Suppression subtractive hybridization (SSH) is frequently used to unearth differentially expressed genes on a whole-genome scale. Its versatility is based on combining cDNA library subtraction and normalization, which allows the isolation of sequences of varying degrees of abundance and differential expression. SSH is a complex process with many adjustable parameters that affect the outcome of gene isolation.We present a mathematical model of SSH based on DNA hybridization kinetics for assessing the effect of various parameters to facilitate its optimization. We derive an equation for the probability that a particular differentially expressed species is successfully isolated and use this to quantify the effect of the following parameters related to the cDNA sample: (a) mRNA abundance; (b) partial sequence complementarity to other species; and (3) degree of differential expression. We also evaluate the effect of parameters related to the process, including: (a) reaction times; and (b) extent of driver excess used in the two hybridization reactions. The optimum set of process parameters for successful isolation of differentially expressed species depends on transcript abundance. We show that the reaction conditions have a significant effect on the occurrence of false-positives and formulate strategies to isolate specific subsets of differentially expressed genes. We also quantify the effect of non-specific hybridization on the false-positive results and present strategies for spiking cDNA sequences to address this problem.


**Comparative and Functional Genomics**
*Comp Funct Genom* 2002; **3**: 405–422.
Published online in Wiley InterScience (www.interscience.wiley.com). **DOI:** 10.1002/cfg.206

Research Article

### A mathematical model for suppression subtractive hybridization

Chetan Gadgil[1], Anette Rink[2], Craig Beattie[2] and Wei-Shou Hu[1]*
[1] *Department of Chemical Engineering and Materials Science, 421 Washington Avenue SE, University of Minnesota, Minneapolis, MN 55455, USA*
[2] *Department of Animal Biotechnology, School of Veterinary Medicine, University of Nevada, Reno, NV 89557, USA*

**Correspondence to: Wei-Shou Hu, Department of Chemical Engineering and Materials Science, University of Minnesota, Minneapolis, MN 55455, USA. E-mail: acre@cems.umn.edu*

Received: 27 February 2002
Accepted: 1 August 2002

### Abstract

**Suppression subtractive hybridization (SSH) is frequently used to unearth differentially expressed genes on a whole-genome scale. Its versatility is based on combining cDNA library subtraction and normalization, which allows the isolation of sequences of varying degrees of abundance and differential expression. SSH is a complex process with many adjustable parameters that affect the outcome of gene isolation. We present a mathematical model of SSH based on DNA hybridization kinetics for assessing the effect of various parameters to facilitate its optimization. We derive an equation for the probability that a particular differentially expressed species is successfully isolated and use this to quantify the effect of the following parameters related to the cDNA sample: (a) mRNA abundance; (b) partial sequence complementarity to other species; and (3) degree of differential expression. We also evaluate the effect of parameters related to the process, including: (a) reaction times; and (b) extent of driver excess used in the two hybridization reactions. The optimum set of process parameters for successful isolation of differentially expressed species depends on transcript abundance. We show that the reaction conditions have a significant effect on the occurrence of false-positives and formulate strategies to isolate specific subsets of differentially expressed genes. We also quantify the effect of non-specific hybridization on the false-positive results and present strategies for spiking cDNA sequences to address this problem. Copyright © 2002 John Wiley & Sons, Ltd.**

**Keywords:** suppression subtractive hybridization; mathematical model; hybridization kinetics

### Introduction

Developmental processes such as ageing, metamorphosis, and embryo development are associated with changes in gene expression (Hill *et al.*, 2000; Lee *et al.*, 1999; White *et al.*, 1999). Cells exposed to different extracellular environments, at different metabolic levels or pathophysiologic states, also exhibit different profiles of gene expression (Alizadeh and Staudt, 2000; Bittner *et al.*, 2000; DeRisi *et al.*, 1997; Oh and Liao, 2000). The transformation of genotype to a variety of phenotypes is characterized by differential gene expression from the same repertoire of sequence information. An important first step in the elucidation of the molecular mechanisms responsible for altered physiological states or developmental pathways is the identification of genes that are differentially regulated at the transcriptional level.

Several methods to profile gene expression have been developed. The expression profile for each sample may be estimated separately and then compared by methods that depend on specific hybridization of probes to DNA microarrays (Lipshutz *et al.*, 1999) or on the counting of tags or signatures of DNA fragments (Brenner *et al.*, 2000; Velculescu *et al.*, 1995). Differences in gene expression between two samples can be compared

Copyright © 2002 John Wiley & Sons, Ltd.

directly by methods such as differential display (Liang and Pardee, 1992), two-colour microarray hybridization (Brown and Botstein, 1999), subtractive cloning techniques (Sagerstrom *et al.*, 1997), and combinations of these (Pardinas *et al.*, 1998; Yang *et al.*, 1999). These approaches have been successfully used to identify genes differentially expressed in two populations that exhibit large changes in expression levels, or genes that are expressed at high concentrations in terms of number of copies per cell. Closed systems such as DNA microarrays require genomic sequence information in order to identify differentially expressed transcripts. Open systems have the flexibility of identifying uncatalogued sequences. However, many techniques have a low efficiency of identifying rare genes that are differentially expressed (Martin and Pardee, 2000). This problem is exacerbated when the change in expression level of rare transcripts is small. Since genes expressed at low levels also play a role in establishing differentiated phenotypes, their identification is essential for a complete mechanistic understanding of cellular changes.

### Suppression subtractive hybridization

Suppression subtractive hybridization (SSH), a technique to identify a set of genes differentially expressed in two cell samples, has the promise of overcoming some of these difficulties (Diatchenko *et al.*, 1996). The singular advantage of SSH lies in the ability to identify differentially expressed genes, irrespective of the level of expression, in the absence of sequence information. SSH has been used to investigate differential expression in a variety of experimental systems, including malignant melanoma (Hipfel *et al.*, 2000), liver regeneration (Groenink and Aad, 1996), embryo development (Simpson *et al.*, 1999) and honeybee larval development (Evans and Wheeler, 1999). SSH identified differentially expressed sequences with no matches in the public databases in all these systems.

The SSH process *normalizes* the levels of rare and abundant genes, and *subtracts* genes expressed in both samples. Genes upregulated in one sample (referred to as tester) relative to the other sample (called the driver) can be identified. The SSH process (Figure 1) entails two rounds of hybridization followed by two PCR reactions (Diatchenko *et al.*, 1996). Poly $A^+$ mRNA is isolated from total RNA and reverse-transcribed to give a double-stranded cDNA pool. The cDNA is digested by *RsaI*, resulting in fragments 0.1–2 kb long. This step reduces the size distribution of cDNA species and creates blunt ends for adaptor ligation. The tester is divided into two equal parts (referred to as tester A and tester B) and ligated with different adaptors (adaptor A and adaptor B) at the 5′ end of each fragment. In the first set of hybridizations (hybridization 1A and hybridization 1B), an excess of driver sample is added to each tester fraction separately, and the reactions are allowed to proceed under identical conditions. Among species present at the same concentration in the tester, those present in similar or higher levels in the driver will form duplexes at a faster rate than those whose concentration in the driver is lower. This leads to an enrichment of single-stranded species that are present at a higher level in the tester. Due to the second-order hybridization process, normalization of the concentration of single-stranded species is also achieved, as abundant species form duplexes at a higher rate than rare species. In the second hybridization, the end products of hybridization 1A and hybridization 1B are mixed and additional excess single-stranded driver is added for further subtraction. Unsubtracted single stranded species from hybridization 1A and hybridization 1B form duplexes in which one strand has adaptor A and the other strand has adaptor B. The duplex species formed during the two hybridization steps are shown in Figure 1.

After the hybridization reactions, end-filling of duplexes with adaptor overhangs is carried out to form blunt-ended DNA. The duplexes are then amplified by PCR using adaptor A and adaptor B as primers. This leads to a differential amplification, depending on the nature of the duplex. Those duplexes in which the two strands have different adaptors are exponentially amplified in the PCR reactions. Duplexes in which both strands have identical adaptors at both ends form panhandle-like structures because of the self-complementary nature of the adaptors and are not amplified. Duplexes with an adaptor only at one end are linearly amplified.

The PCR products are then ligated into vectors that are used to transform *Escherichia coli*. In a successful SSH the frequency of the sequences isolated from *E. coli* clones is greater for genes that are expressed at a higher level in the tester. To identify genes that are downregulated in the sample

Copyright © 2002 John Wiley & Sons, Ltd.

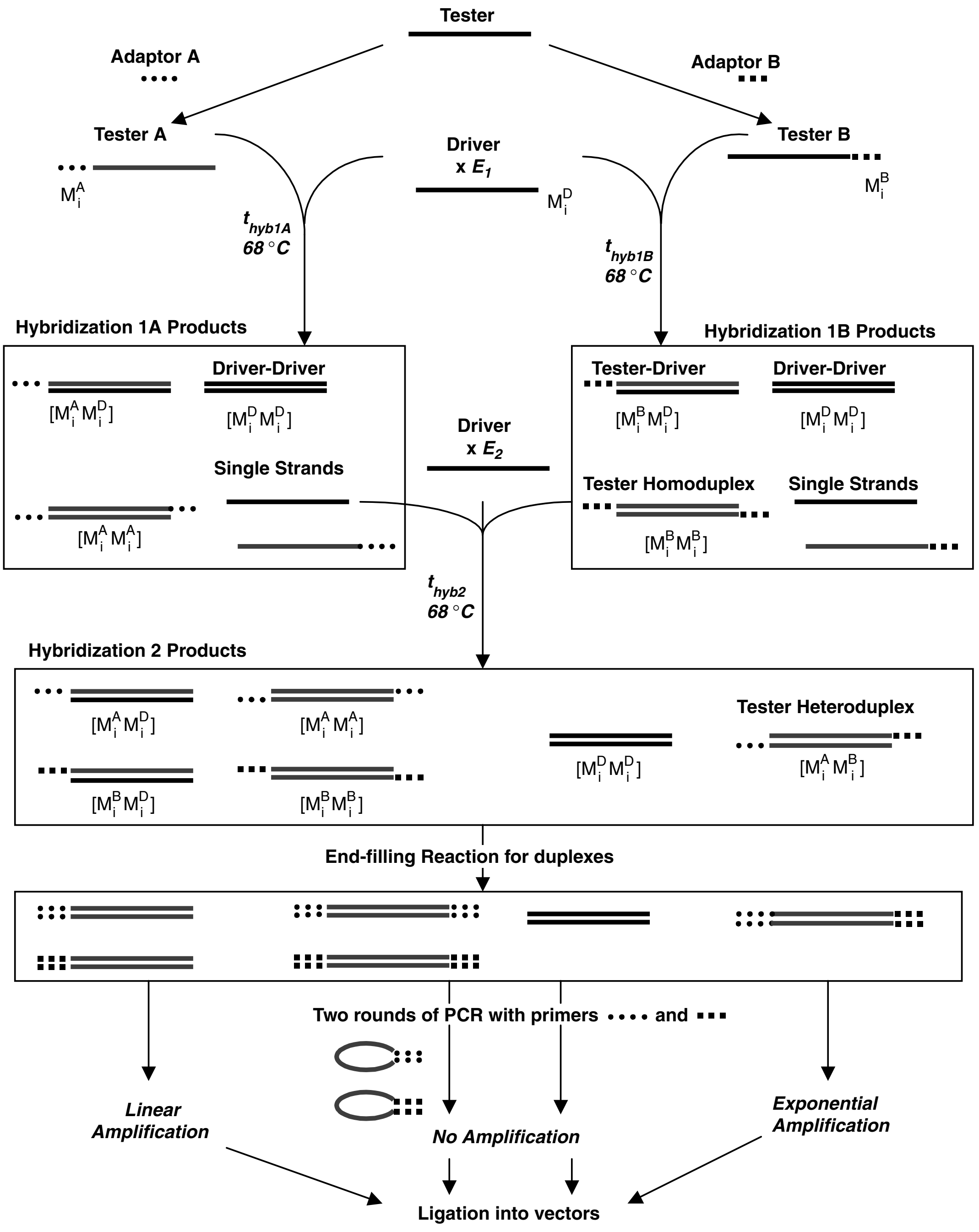

**Figure 1.** Schematic of the SSH process depicting the cDNA species formed during the hybridization reactions used as the tester, a reverse SSH is carried out by switching the samples used as tester and driver.

SSH accomplishes normalization and subtraction by taking advantage of the different rates of hybridization of cDNA strands for different genes, depending on their abundance level and the degree of (differential) expression. The extent of hybridization is governed by the hybridization temperature, hybridization times and the driver : tester ratio. The effect of these operating parameters on the efficiency of normalization and subtraction, and thus the probability that a particular differentially

Copyright © 2002 John Wiley & Sons, Ltd. 

expressed gene is isolated, differs depending on each gene's abundance level and extent of differential expression. As the conditions that lead to the highest probability of successful isolation vary with the abundance, level of differential expression, length of the transcript and degree of sequence similarity to other transcripts, there may not be a unique optimal condition of SSH for isolating all differentially expressed genes. The large number of parameters that affect the outcome of SSH suggest the use of a mathematical model to facilitate the selection of experimental conditions.

### Mathematical models for subtractive hybridization

Attempts to model various techniques of isolating differentially expressed transcripts have assumed DNA hybridization to occur as a simple irreversible bimolecular reaction, and used the hybridization model developed by Wetmur and co-workers (Wetmur, 1976; Wetmur and Davidson, 1968). Ermolaeva *et al.* (1996; Ermolaeva and Wagner, 1995) developed a mathematical model for a subtractive hybridization process where the tester contains only three differentially expressed transcripts, which are completely absent in the driver, and presented an analytical solution for the case of large driver excess. The subtractive hybridization model of Cho and Park (1998) explored the effect of differing tester : driver ratios on target identification in cases where target sequences present in the tester are totally absent in the driver. Milner *et al.* (1995) have presented a model for subtractive hybridization of genomic deletion mutants and enrichment of upregulated sequences, and predicted target enrichment in genomic subtractions consistent with experimental results. This model, however, does not present an analysis of the probability of isolation of a particular differentially expressed sequence.

Since it is more likely that genes are differentially regulated from a basal level rather than being switched on or off (Gurskaya *et al.*, 1996), a mathematical model for a process that identifies sequences differentially expressed between two cell samples should consider all possible levels of regulation. Microarray experiments and studies using serial analysis of gene expression (SAGE) (Zhang *et al.*, 1997) have clearly demonstrated a range in the level of gene expression, with the majority of genes exhibiting limited if not negligible differential expression (Sagerstrom *et al.*, 1997). Therefore, any attempt to model the SSH process should also consider the concentrations of non-differentially expressed species that remain at the end of the process. This concentration can then be used to estimate the number of false-positives, and the probability that the process will isolate a particular differentially expressed species. In a cDNA pool, there exist several sequences that have a partial sequence homology to each other. The formation of chimeric cDNAs during the SSH process has been observed (Zhang *et al.*, 2000). A comprehensive mathematical model for a process based on DNA hybridization should account for non-specific hybridizations between strands that have partial sequence homology.

In this report, we present a model for the SSH process that enables analysis of the effects of process variables, such as the amount of driver excess and the hybridization times on the probability of identifying differentially expressed cDNA species having a certain abundance, relative expression level, and degree of sequence similarity to other sequences in the hybridization mix.

### Model development

We represent single-stranded cDNA for species $i$ ($i = 1, 2, \ldots n_T$) by $M_i^A$, $M_i^B$ or $M_i^D$ where the superscript denotes the type of adaptor (adaptor A, adaptor B, or no adaptor) and $n_T$ is the total number of distinct sequences. After hybridization reactions, each single-stranded species $M_i^p$ (p = A, B or D) may form three types of duplexes $M_i^p M_j^q$: (a) homo-duplexes with complete sequence match (i = j) and with the same adaptors on both strands or both strands from the driver (p = q); (b) homoduplexes of strands that are perfectly complementary (i = j) except for the adaptors ($p \neq q$); and (c) heteroduplexes of partially complementary strands ($i \neq j$). We assume that the behaviour of the sense and antisense strands of each species will be completely symmetrical. Therefore, the model focuses on one strand.

The mass balance equations for the single strands $M_i^p$ and duplex $M_i^p M_j^q$ during the hybridization reactions are given by Equations 1 and 2,

Copyright © 2002 John Wiley & Sons, Ltd. 

respectively:

$$\frac{d\,[M_i^p]}{dt} = -\sum_{q=A,B,D}\ \sum_{j=1}^{n_T}(kf_{i,j}^{p,q}[M_i^p][M_j^q]) + \sum_{q=A,B,D}\ \sum_{j=1}^{n_T}(kb_{i,j}^{p,q}[M_i^p M_j^q]) \quad (1)$$

$$\frac{d\,[M_i^p M_j^q]}{dt} = \frac{1}{1+\delta_{ij}\delta_{pq}}(kf_{i,j}^{p,q}[M_i^p][M_j^q] - kb_{i,j}^{p,q}[M_i^p M_j^q]) \quad (2)$$

where $\delta$ is the Kroneker delta function ($\delta = 1$ if subscripts are equal, 0 otherwise). The first term in each equation represents duplex formation (each species $M_i^p$ may react with all other single strands $M_j^q$ to form the corresponding duplex $M_i^p M_j^q$) and the second term represents the reverse reaction, i.e. duplex melting. Such equations are formulated for all species and solved simultaneously to obtain the concentrations of various single-stranded and duplex molecules during the course of the hybridization reactions.

### Model development for ideal hybridization

In our initial analysis, it is assumed that only perfectly complementary strands react irreversibly to form duplexes (i.e. $kf_{i,j}^{p,q} = 0$ for $i \neq j$ and $kb_{i,j}^{p,q} = 0 \forall i,j$). The rate constant for the duplex formation reaction is assumed to be the same for all species, i.e. $kf_{i,i}^{p,q} = \text{constant} = k_f$. Initial concentrations for the single-stranded and duplex species at the beginning of the hybridization reactions are given in Table 1. Using these assumptions to simplify Equations 1 and 2, the mass balance equations for each step of the hybridization processes are formulated and listed in the Appendix. The concentration of duplexes with two different adaptors, A and B, at the end of the hybridization process can be expressed in terms of the initial concentration of the single-stranded species $[M_i^A]_{0,1}$, the relative expression ratio $\kappa_i$, the hybridization rate constant $k_f$ and the hybridization time ($t_{hyb1}, t_{hyb2}$) and driver excess ($E_1, E_2$) for the two reactions as:

$$[M_i^A M_i^B]|_{t_{hyb2}} = \frac{[M_i^A]_{0,1}^2 k_f t_{hyb2}\kappa_i^3}{6(2\kappa_i + [M_i^A]_{0,1}\ k_f\ t_{hyb1}(E_1+\kappa_i))([M_i^A]_{0,1}^2 \times E_2 t_{hyb1} t_{hyb2}(E_1+\kappa_i)\ k_f^2 + [M_i^A]_{0,1}\kappa_i \times (3(E_1+\kappa_i)\ t_{hyb1} + 2(E_1+E_2+\kappa_i) \times\ t_{hyb2})\ k_f + 6\kappa_i^2)} \quad (3)$$

The duplex concentration remains unchanged during the end-filling reaction. During the final PCR reaction, species are amplified exponentially, amplified linearly, or not amplified depending on the nature of the adaptors. After the PCR step, the

Table 1. Initial conditions for hybridization reactions

| Species | Hybridization 1A | Hybridization 1B | Hybridization 2 |
|---|---|---|---|
| $[M_i^A]$ | $[M_i^A]_{0,1} = \frac{[M_i^A]_{tester}}{2}$ | 0 | $\frac{[M_i^A]^*}{3}$ |
| $[M_i^B]$ | 0 | $\frac{[M_i^A]_{tester}}{2}$ | $\frac{[M_i^A]^*}{3}$ |
| $[M_i^D]$ | $\frac{[M_i^A]_{tester}}{2\kappa_i}E_1$ | $\frac{[M_i^A]_{tester}}{2\kappa_i}E_1$ | $\frac{2\frac{[M_i^D]_{0,1}}{\kappa_i}E_2 + 2\,[M_i^D]^*}{3}$ |
| $[M_i^A M_i^A]$ | 0 | 0 | $\frac{[M_i^A M_i^A]^*}{3}$ |
| $[M_i^B M_i^B]$ | 0 | 0 | $\frac{[M_i^A M_i^A]^*}{3}$ |
| $[M_i^D M_i^D]$ | 0 | 0 | $\frac{2\,[M_i^D M_i^D]^*}{3}$ |
| $[M_i^A M_i^D]$ | 0 | 0 | $\frac{[M_i^A M_i^D]^*}{3}$ |
| $[M_i^B M_i^D]$ | 0 | 0 | $\frac{[M_i^B M_i^D]^*}{3}$ |
| $[M_i^A M_i^B]$ | 0 | 0 | 0 |

* Concentration at the end of hybridization 1, calculated from equations A.6–A.10.

Copyright © 2002 John Wiley & Sons, Ltd.

total DNA corresponding to gene *i* available for ligation is:

$$[D_i] = [M_i^A M_i^B]|_{t_{hyb2}} \times 2^{n_{PCR}} + ([M_i^A M_i^D]|_{t_{hyb2}} + [M_i^B M_i^D]|_{t_{hyb2}}) \times n_{PCR} + [M_i^A]|_{t_{hyb2}} + [M_i^B]|_{t_{hyb2}} \quad (4)$$

In a typical SSH, the number of PCR cycles ($n_{PCR}$) is high, and hence $2^{n_{PCR}} \gg n_{PCR}$. The right-hand side of Equation 4 is dominated by the first term, and can be expressed as:

$$[D_i] = [M_i^A M_i^B]|_{t_{hyb2}} \times 2^{n_{PCR}} \quad (5)$$

After the PCR reaction, a subtracted cDNA library is constructed and $N_{col}$ colonies are picked for further analysis as putative differentially expressed genes. The probability $p_s$ that species s is among those $N_{col}$ colonies depends on the fraction ($f_s$) of the PCR product corresponding to species s. The probability that, of $N_{col}$ colonies, none corresponds to species s is $(1 - f_s)^{N_{col}}$. Hence, the probability that at least one colony corresponds to s is:

$$p_s = 1 - (1 - f_s)^{N_{col}} \quad (6)$$

where $f_s$ is the ratio of the DNA concentration corresponding to species *s* available for ligation to the total DNA concentration for all $n_T$ species available for ligation:

$$f_s = \frac{D_s}{\sum_{i=1}^{n_T} D_i} \quad (7)$$

Substituting Equation 5 in Equation 7, we get the expression for the probability of identification of species *s* for ideal hybridizations as:

$$f_s = \frac{[M_s^A M_s^B]|_{t_{hyb2}}}{\sum_{i=1}^{n_T} [M_i^A M_i^B]\Big|_{t_{hyb2}}} \quad (8)$$

The mathematical framework outlined here provides an analytical expression for the concentration of the different duplex species that are formed after the two hybridization steps as a function of the initial concentrations of the cDNA single strands and the reaction conditions (Equation 3). Substituting this expression in Equation 8, the probability of isolation of a particular differentially expressed gene can be obtained.

### Non-specific hybridization of partially complementary strands

To account for non-specific hybridizations in which single strands that are not perfectly complementary hybridize to form heteroduplexes of the type $M_i^p M_j^q$ ($i \neq j$), we consider the case where there exist two species, i and j, with varying degrees of sequence complementarity. Equations 1 and 2 are solved simultaneously to simulate the system. If the number of species that have partially complementary sequences is $N_s$, it can be shown that we have to solve 4.5 $N_s$ ($N_s + 1$) simultaneous ordinary differential equations to fully simulate the system. Since this number scales as the square of the species involved, the problem quickly becomes computationally intractable with just a few species.

Through numerical simulations of these model equations, the concentration of duplex species that are formed from the hybridization of one strand with adaptor A with another strand with adaptor B ($[M_i^A M_j^B]$) can be simulated. An assumption is made that such a heteroduplex is not dissociated during the end-filling step. The heteroduplexes will then be amplified by the PCR process. The total amount of DNA available for ligation after the PCR steps, given for ideal hybridizations by Equation 4, can be rewritten for this situation as:

$$[D_i] = \left( \sum_{j=1}^{N_s} [M_i^A M_j^B]\Big|_{t_{hyb2}} + [M_i^A M_i^B]\Big|_{t_{hyb2}} \right) \times 2^{n_{PCR}} + \left( \sum_{j=1}^{N_s} [M_i^A M_j^D]\Big|_{t_{hyb2}} + \sum_{j=1}^{N_s} [M_i^B M_j^D]\Big|_{t_{hyb2}} \right) \times n_{PCR} + [M_i^A]\Big|_{t_{hyb2}} + [M_i^B]\Big|_{t_{hyb2}} \quad (9)$$

As $2^{n_{PCR}} \gg n_{PCR}$, the value of $D_i$ can be approximated as:

$$[D_i] = \left( \sum_{j=1}^{N_s} [M_i^A M_j^B]\Big|_{t_{hyb2}} + [M_i^A M_i^B]\Big|_{t_{hyb2}} \right) \times 2^{n_{PCR}} \quad (10)$$

Copyright © 2002 John Wiley & Sons, Ltd.

This value can then be substituted in Equation 7 to obtain:

$$f_s = \frac{\sum_{j=1}^{N_s}[M_i^A M_j^B]\Big|_{t_{hyb2}} + [M_i^A M_i^B]\Big|_{t_{hyb2}}}{\sum_{i=1}^{n_T}\left(\sum_{j=1}^{N_s}[M_i^A M_j^B]\Big|_{t_{hyb2}} + [M_i^A M_i^B]\Big|_{t_{hyb2}}\right)} \quad (11)$$

From the numerically computed values of $[M_i^A M_j^B]|_{t_{hyb2}}$, Equation 11 can be used to calculate the fraction of DNA corresponding to the species *i* and hence the probability of identification of at least one colony containing the sequence can be estimated using Equation 6.

### Simulation parameters

To use the developed equations for simulation of the SSH process, the reaction rate constants and initial concentrations of $M_i$ have to be determined. The cDNA is digested with *RsaI*, leading to single strands with an average molecular mass of ~150 kDa, corresponding to a length of 470 bp. The experimentally observed range of strand lengths is 200–2000 bp. Using a total cDNA concentration in the tester of 2 μg cDNA and taking reagent dilution into account, the total cDNA concentration is calculated to be $1 \times 10^{-7}$ M. Poly$A^+$ mRNA in a typical mammalian cell is divided into three abundance classes: abundant, intermediate and rare species (Hastie and Bishop, 1976). The number of species in each class and their relative abundance is shown in Table 2. This classification is also computationally convenient as it enables the estimation of the average initial concentration of a species in a particular class from the total cDNA concentration (Table 2).

Table 2. Abundance classes of mRNA in a typical mammalian cell

| mRNA Class | Number of sequences | Abundance (copies/cell) | Conc. of each in tester |
|---|---|---|---|
| Abundant | 10 | 12 500 | $2.5 \times 10^{-9}$ M |
| Intermediate | 750 | 300 | $5.5 \times 10^{-11}$ M |
| Rare | 12 000 | 15 | $3 \times 10^{-12}$ M |

The concentrations of species $M_i$ in the driver are the corresponding tester concentrations multiplied by the excess ratio E and divided by the differential expression ratio $\kappa_i$. As equal volumes of tester and driver are mixed at the start of the hybridization process, the initial concentration of tester and driver species in the hybridization mixture is half that in each fraction. The second order rate constant ($k_f$) was taken to be $1 \times 10^6$ $M^{-1}s^{-1}$ (Craig *et al.*, 1971; Ermolaeva and Wagner, 1995). The renaturation rate constant for non-specific hybridization depends on the percentage sequence identity. Vernier and co-workers (Vernier *et al.*, 1996) report that the values of the renaturation rate constant decreases to 98%, 80%, and 77% of the rate for renaturation of perfectly complementary strands, respectively, for sequences sharing 94%, 83% and 77% sequence identity. These values are used for simulation of the association rates for non-specific hybridization. Anderson and Young (1985) report that the duplex dissociation rate increases by a factor of two for every 10% mismatch of the single-strand sequences. Based on this data and results on melting of chimeric duplexes reported elsewhere (Gotoh *et al.*, 1995; Spiegelman *et al.*, 1973), the values of the dissociation constant for 500 bp strands having partial homologies of 94% and 77% have been estimated as $1 \times 10^{-5}$ $s^{-1}$ and $5 \times 10^{-3}$ $s^{-1}$, respectively, and used for simulating non-specific DNA hybridization.

The DNA hybridization process is never complete for finite hybridization times, and there is a finite probability of isolating a species that is not differentially expressed. The denominator of Equation 8 is the sum of the concentrations of duplexes of the type $M_i^A M_i^B$. Some of these duplexes correspond to cDNA present in a higher concentration in the tester and others represent cDNA present in equal or lower concentration in the tester than the driver. The latter category of genes can lead to false-positive results. To assess the probability of obtaining false-positive results, we divide the total number of genes $n_T$ into genes that are differentially expressed ($n_A$, $n_I$ and $n_R$, corresponding to differentially expressed abundant, intermediate and rare species, respectively), and genes that are not differentially expressed ($n_A^*$, $n_I^*$ and $n_R^*$). The duplexes with different adaptors but from genes that are not differentially expressed ($M_{i*}^A M_{j*}^B$) give rise to false-positive results. The fraction of the

Copyright © 2002 John Wiley & Sons, Ltd. 

tester homoduplex with different adaptors A and B on the two strands for a particular species *s* (Equation 8) can be rewritten as:

$$f_s = \frac{[M_s^A M_s^B]}{\sum_{i=1}^{n_T} [M_i^A M_i^B]}$$

$$= \frac{[M_s^A M_s^B]}{n_R^*[M_{R*}^A M_{R*}^B] + n_I^*[M_{I*}^A M_{I*}^B] + n_A^*[M_{A*}^A M_{A*}^B] + n_R[M_R^A M_R^B] + n_I[M_I^A M_I^B] + n_A[M_A^A M_A^B]}$$

$$= \frac{[M_s^A M_s^B]}{baseline + n_R[M_R^A M_R^B] + n_I[M_I^A M_I^B] + n_a[M_a^A M_a^B]};$$

$$baseline = n_R^*[M_{R*}^A M_{R*}^B] + n_I^*[M_{I*}^A M_{I*}^B] + n_A^*[M_{A*}^A M_{A*}^B] \quad (12)$$

The value *baseline* is the total concentration of tester homoduplex with different adaptors A and B on the two strands from genes that are not differentially regulated. These sequences lead to the formation of false-positives. The value of the baseline depends on the number of genes whose expression levels in the tester and driver are the same. A survey of the literature reveals a large variation in the number of differentially expressed genes among samples from different sources. Expression analysis of 8740 rat genes using high-density DNA array technology revealed that 873 genes exhibit statistically significant differences in gene expression levels during nephrogenesis (Stuart *et al.*, 2001). An analysis of publicly available microarray data (**http://ep.ebi.ac.uk/EP/EPCLUST/**) shows that approximately 20% of genes selected for microarray construction to investigate differences in gene expression between normal and cancer cells were differentially expressed. In closely related cell types (B and T lymphocytes), 2% of the genes were found to be differentially expressed using a subtractive hybridization approach (Sagerstrom *et al.*, 1997). In a survey of the whole genome using SAGE, approximately 1.5% of expressed genes were found to be unequally expressed in normal and cancer cells (Zhang *et al.*, 1997).

Like SAGE, SSH is an open system where all expressed transcripts are probed to isolate differentially expressed genes. We are interested in the study of cells in different metabolic states and spheroid formation in hepatocytes, i.e. probing differences in closely related cell types. Hence we assume that ∼1.5% of genes will be differentially expressed and estimate the number of genes that are not differentially expressed. We have assumed that 11 800 rare, 740 intermediate and 10 abundant genes are present in equal concentrations in the tester and driver samples, i.e. $n_{R*} = 11800$, $n_{I*} = 740$ and $n_{A*} = 10$. These values are used to calculate the *baseline* concentration.

All symbolic calculations of partial derivatives were carried out using Mathematica™ 4.0 (Wolfram Research, Champaign, IL). All numerical calculations were carried out using Matlab™ 5.3.1 (The MathWorks, Natick, MA).

### Results

Equation 3 is used to estimate the concentration of tester homoduplex with different adaptors on the two strands $M_i^A M_i^B$ relative to the total concentration of false-positives, and to investigate the effect of a change in the reaction conditions. The baseline value varies with the conditions used for SSH. For easy comparison among different conditions, the concentrations $M_i^A M_i^B$ are normalized to the baseline and denoted as $\Psi_i$. A large value of $\Psi_i$ ($\Psi_i \gg 1$) indicates a high likelihood of isolating a clone corresponding to species *i*. Conversely, a lower $\Psi_i$ represents a high likelihood that a large number of false-positive clones will be obtained before species *i* is isolated. The results shown are for strands of various initial concentrations corresponding to rare, intermediate-abundance, and abundant mRNA. Results are shown for levels of differential regulation corresponding to $\kappa_i = 1$, 10, 100 and 1000. The initial concentration in the driver sample is calculated by dividing the initial concentration in the tester sample by $\kappa_i$. As the number of sequences that are not differentially expressed does not change in the reverse subtraction process, the results for an abundant species with $\kappa_i = 1000$ correspond to both an abundant sequence that is downregulated 1000-fold, and a rare sequence that is upregulated 1000-fold.

### Ideal hybridization

Figure 2 shows the normalized concentration ($\Psi_i$) of $M_i^A M_i^B$ duplexes at the end of the second

Copyright © 2002 John Wiley & Sons, Ltd.

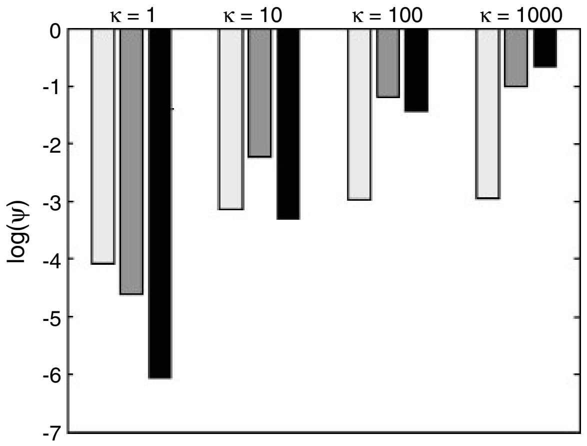

**Figure 2.** Ratio of concentration duplex with different adaptors A and B on each strands to concentration of baseline ($\psi$) for sequences belonging to the abundant (■), intermediate-abundance (▩) and rare (□) genes as a function of the relative differential expression ratio ($\kappa$). Simulations are carried out for $t_{hyb1} = 8h$, $t_{hyb2} = 12h$, and $E_1 = E_2 = 30$

hybridization as a function of the relative differential expression ($\kappa_i$) for values of the reaction parameters recommended by the Clontech PCR-Select™ protocol ($E = 30$, $t_{hyb1} = 8$ h, $t_{hyb2} = 12h$) (Clontech Manual, 1999). It is seen from the figure that the value of $\Psi_i$ for differentially expressed species is less than 1 (i.e. <0 in the log scale, as shown in Figure 2) for all abundance classes. In other words the probability of obtaining a false-positive clone is higher than that of obtaining a particular true positive. As is seen from the graph, the value of $\Psi_i$, and hence the probability that a particular species $i$ is identified by the SSH procedure, depends greatly on $\kappa_i$ and the initial concentration. For example, for abundant species that are not differentially expressed ($\kappa_i = 1$), the value of $\Psi_i$ is much lower than that of rare species with $\kappa_i = 1$. Thus, in the absence of non-specific hybridization, abundant species are efficiently eliminated by the SSH process under recommended process conditions. This leads to the conclusion that under these conditions, the bulk of the false-positive sequences resulting from the SSH process will consist of rare sequences that are not differentially expressed. However, for abundant sequences that are downregulated by a large extent ($\kappa_i = 1000$), the value of $\Psi_i$ is close to one, and higher than the corresponding value for intermediate and rare sequences. This implies that the SSH process is biased towards sequences that are differentially expressed to a large extent. However, there is poor efficiency of identifying rare sequences that are downregulated even further. Even an on–off regulation of rare sequences (as approximated by the bar corresponding to rare sequences with $\kappa_i = 1000$) does not lead to an improvement in the efficiency. For moderate differential expression levels ($\kappa_i = 10-100$), the SSH process is most successful in identifying rare sequences that are upregulated 100-fold, or intermediate abundance sequences that are downregulated 100-fold.

The effect of hybridization times and excess ratios on the relative concentrations of the $M_i^A M_i^B$ duplex was evaluated by varying the value of one reaction parameter while keeping all the others constant. The normalized partial derivative of $\Psi_i$ with respect to the parameter being changed, $Z_{parameter} = \frac{(parameter)}{\Psi_i} \frac{\partial \Psi_i}{\partial (parameter)}$, describes how the probability of isolating a true differentially expressed transcript varies with a change in the parameter. A value of $Z = +1$ implies a 100% increase in the relative concentration of $M_i^A M_i^B$ to baseline due to a 100% increase in the parameter value. Shown in Figure 3 are plots of such partial derivatives with respect to the excess ratios ($E_1$ and $E_2$) and reaction times ($t_{hyb1}$ and $t_{hyb2}$) for the first and second hybridization as a function of $\kappa_i$. Figure 3a shows that, except for transcripts with a low ($\kappa_i < 20$) level of differential expression, $Z_{E1}$ is positive, and therefore increasing $E_1$ is beneficial as it results in an increase in the $\Psi_i$ corresponding to differentially expressed species and leads to fewer false-positives.

The effect of changing $E_2$ on the relative $M_i^A M_i^B$ concentration is shown in Figure 3b. For rare transcripts, $Z_{E2} > 0$ for all values of $\kappa_i$, and the value of $\Psi_i$ increases with an increase in the excess ratio for hybridization 2. However, for abundant transcripts, the trend is opposite, i.e. $\Psi_i$ decreases as the relative concentration as $E_2$ is increased from a value of 30. For intermediate-abundance species, the effect of changing $E_2$ depends on the degree to which they are differentially expressed ($\kappa_i$). There is a beneficial effect for highly upregulated species ($\kappa_i > 30$) but a negative effect on species with a lower differential expression ratio. The magnitude of this increase is not high (<0.5), showing that the number of false-positives is not very sensitive

Copyright © 2002 John Wiley & Sons, Ltd. 

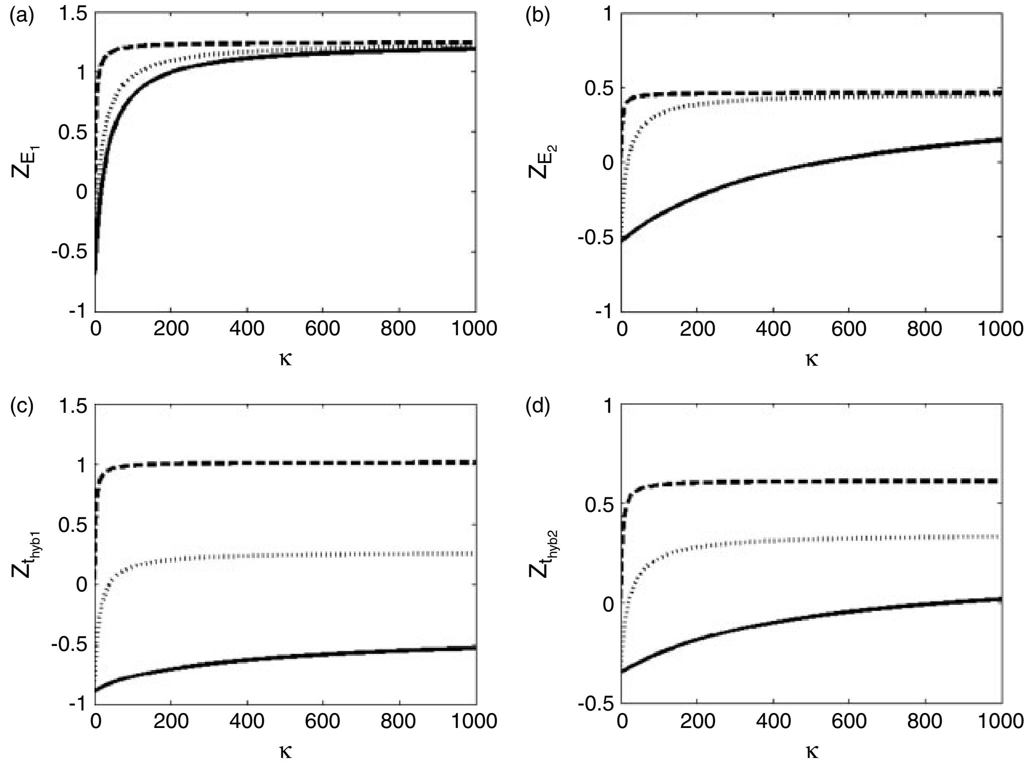

**Figure 3.** Normalized partial derivatives (Z) of the duplex : baseline ratio with respect to process parameters process conditions $t_{hyb1}$, $t_{hyb2}$, $E_1$ and $E_2$ plotted as a function of the differential expression ratio ($\kappa$). The three lines in each plot represent abundant (———) intermediate abundance (· · · · ), and rare (- - - - ) species to changes in $E_2$ from the value of 30. Thus, doubling the excess ratio will increase the relative concentration of rare transcripts by a maximum of 50% from their original level. Increasing the ratio beyond this value is impractical in situations with a constraint on the amount of cDNA available for analysis.

Figures 3c and 3d are plots of the normalized partial derivatives with respect to $t_{hyb1}$ and $t_{hyb2}$. Increasing $t_{hyb1}$ leads to an improvement in $\Psi_i$ for rare species and a decrease in $\Psi_i$ for differentially expressed abundant species. For intermediate abundance species with a low $\kappa_i$ ($<50$), the effect of increasing $t_{hyb1}$ is to decrease $\Psi_i$, but for highly upregulated intermediate abundance species, there is a small increase in $\Psi_i$ with increase in $t_{hyb1}$. A similar effect is seen from the plot of the normalized partial derivative with respect to $t_{hyb2}$ (Figure 3d), except that the magnitude is much smaller, indicating that the relative concentration ($\Psi_i$) is less sensitive to changes in $t_{hyb2}$ from its value of 12h.

Significantly larger excess ratios are used in other subtractive hybridization based processes such as cDNA-representational difference analysis (cDNA-RDA; Hubank and Schatz, 1994) and normalized library construction (Ko, 1990; Patanjali, 1991; Sasaki *et al.*, 1994). Simulation results for SSH with hybridization times and excess ratios similar to those used in these procedures are presented in Figure 4. The effect of significant decreases in hybridization time and excess ratios are also presented. The beneficial effect of increasing the excess ratios on $\Psi_i$ for differentially expressed species irrespective of abundance levels is clear from the increase observed, even at a low hybridization time of 15 min. On the other hand, increasing $t_{hyb1}$ and $t_{hyb2}$ alone does not yield a significant improvement in $\Psi_i$. The graphs illustrate the beneficial effect of decreasing $t_{hyb1}$ and $t_{hyb2}$ for better identification of differentially expressed abundant species, and increasing $t_{hyb1}$ and $t_{hyb2}$ for better detection of differentially expressed rare species. Similar calculations for other combinations

Copyright © 2002 John Wiley & Sons, Ltd. 

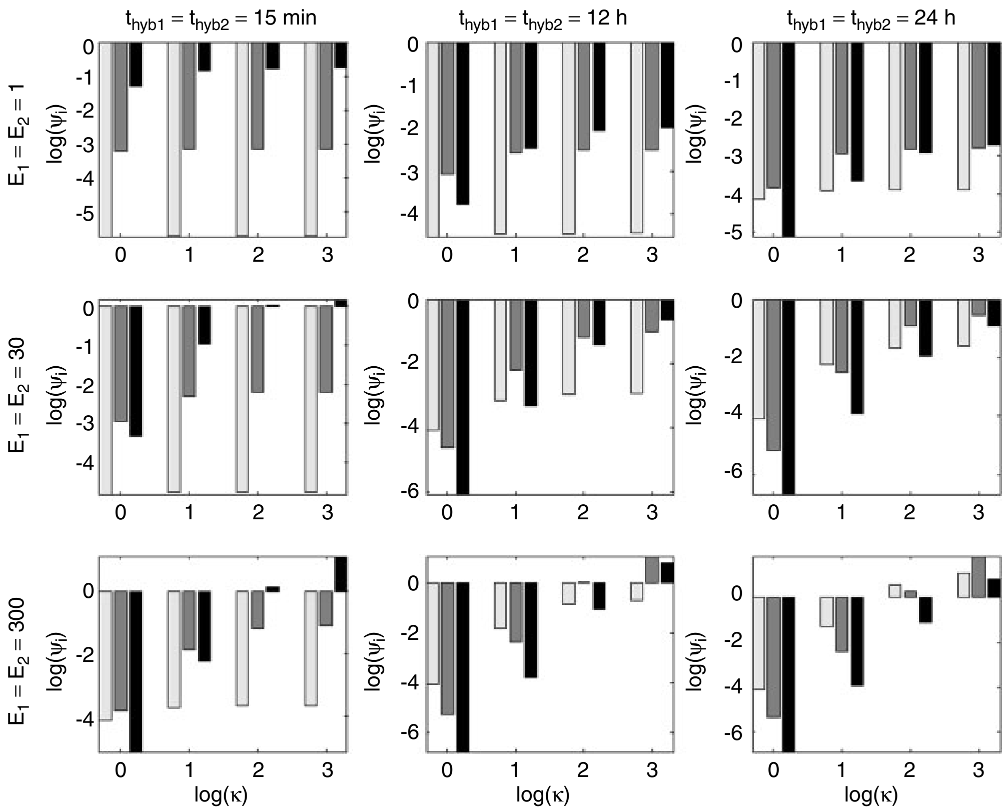

**Figure 4.** Effect of large changes in excess ratios and hybridization times on $\psi$ for abundant (■), intermediate-abundance (■), and rare (□) genes as a function of the relative differential expression ratio ($\kappa$)

of reaction conditions can be easily carried out using this model.

### Effect of non-specific hybridization

The effect of non-specific hybridization is explored using this model. The analysis presented for ideal hybridization can be repeated for conditions where the presence of partially complementary sequences leads to the formation of chimeric duplexes (heteroduplexes). These sequences may each belong to different abundance classes and have different $\kappa$ values. Here we present a detailed analysis of one possible situation where both non-specifically interacting sequences $M_\alpha$ and $M_\beta$ are not differentially expressed ($\kappa_\alpha = \kappa_\alpha = 1$) and hence contribute to the false-positives obtained in the SSH process. Thus, the objective of changing process conditions here is to minimize $\Psi_\alpha$ and $\Psi_\beta$, or at least to reduce those to values corresponding to the false-positives for ideal hybridizations ($\Psi_i$ for sequences with $\kappa_i = 1$). In the following discussion, subscripts $\alpha$ and $\beta$ are used to represent sequences with partial sequence similarity.

Figure 5a shows the effect of the presence of an abundant species, $M_\alpha$, with 94% sequence homology to another abundant sequence, $M_\beta$, on the false-positives that are obtained. The false-positives that are obtained in the absence of non-specific hybridization are also shown for comparison. As seen in Figure 5a, in the presence of non-specific hybridization, the concentration of abundant sequences that are not differentially expressed ($\kappa_\alpha = 1$) is much higher than that of rare sequences with $\kappa_\alpha = 1$. This is the exact opposite of the case where there is no non-specific interaction, where the concentration of false-positive sequences that are abundant is much lower than that of rare sequences. It is also seen that in the presence of non-specific hybridization, $\Psi_\alpha$ for an abundant sequence with $\kappa_\alpha = 1$ is more than 100 times greater than the $\Psi_i$ value for any rare species with $\kappa_i = 1$ in the absence of non-specific interactions.

Copyright © 2002 John Wiley & Sons, Ltd. 

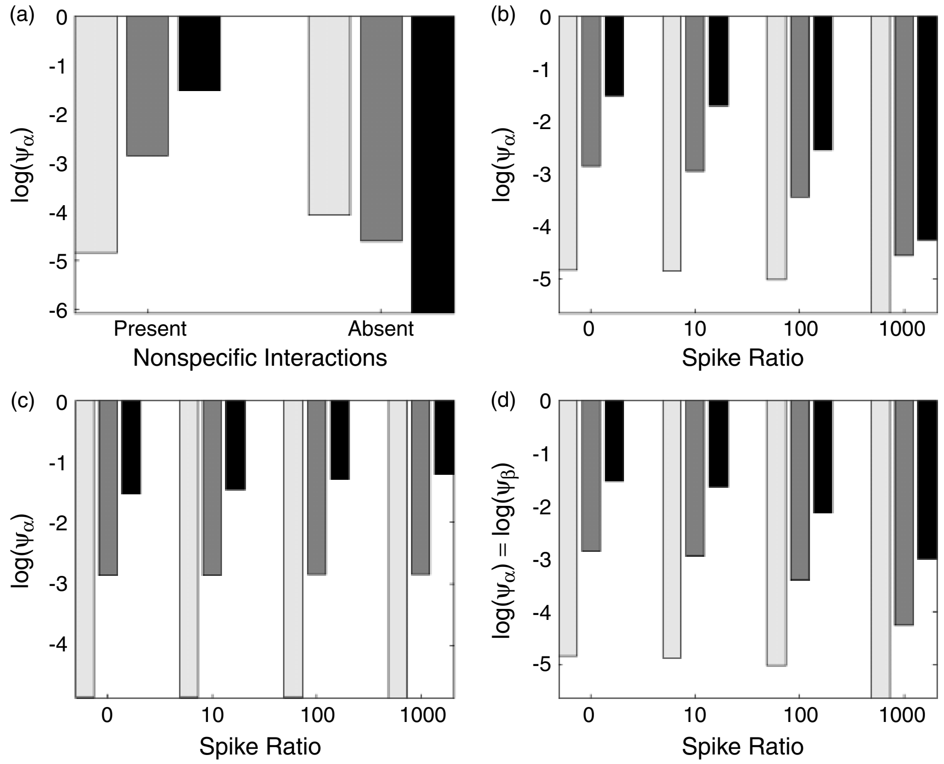

**Figure 5.** Effect of non-specific interactions on SSH performance. (a) Effect on the nature of false-positives of the presence and absence of non-specific hybridizations. (b) Effect of spiking one of the interacting species $\alpha$ on $\Psi_\alpha$. (c) Effect of spiking one of the interacting species $\beta$ on the other species $\Psi_\alpha$. (d) Effect of spiking both interacting species $\alpha$ and $\beta$ on $\Psi_\alpha$ (or $\Psi_\beta$). Figures show $\psi$ for abundant (■), intermediate-abundance (■) and rare (□) genes. Simulations are carried out for $t_{hyb1} = 8h$, $t_{hyb2} = 12h$, and $E_1 = E_2 = 30$

The simulation results presented in Figure 5a show that the false-positive pool obtained will be dominated by such an abundant sequence with $\kappa_\alpha = 1$ and partial homology to another abundant sequence. It should be noted that although results shown in this figure are for strands corresponding to the sequence $M_\alpha$, both the sequences $M_\alpha$ and $M_\beta$ that are partially complementary are affected the same way during the SSH process if they are both present in the same concentrations in the tester and driver. Hence, any analysis presented here for the sequence $M_\alpha$ is equally applicable to $M_\beta$ and both sequences will occur to the same extent in the false-positive pool.

Non-specific hybridization results in false-positive sequences and decreased probability of isolating genes that are truly differentially expressed. This masking effect of partially complementary, abundant sequences can be reduced by increasing the subtraction efficiency through addition of the specific sequence $M_\alpha$ to the driver. The increase in the concentration of the driver strands for that sequence increases the rate of formation of tester–driver duplexes. The effect of such spiking is shown in Figure 5b. The figure depicts $\Psi_\alpha$ values that are obtained when the driver is spiked with increasing amounts (no spiking, 10, 100 and 1000-fold spiking) of the sequence $M_\alpha$. It is seen that there is a beneficial effect of spiking $M_\alpha$. At a level of a 1000-fold spiking, $\Psi_\alpha$ decreases to a level comparable to that in the absence of non-specific interaction.

As indicated above, there is a symmetrical relationship between species $M_\alpha$ and $M_\beta$ allowing us to interpret these results for the sequence $M_\beta$ as well. Thus, when $M_\beta$ is spiked, $\Psi_\beta$ will decrease. However, the effect of spiking one sequence (say $M_\alpha$) on false-positives arising from another non-specifically interacting sequence ($M_\beta$) may not always be positive. Such effects are simulated using

Copyright © 2002 John Wiley & Sons, Ltd.

the model and results are presented in Figure 5c, which shows the effects of spiking $M_\beta$ on $\Psi_\alpha$. The values of $\Psi_\alpha$ for species of different abundance levels and $\kappa_\alpha = 1$ either remains the same or increases with increasing spiking of $M_\beta$. Thus, the simulation results show that there is a detrimental effect on false-positives arising from $M_\alpha$ when $M_\beta$ is spiked at increasing levels. With an increase in the amount of $M_\beta$ that is spiked, the concentration of $M_\alpha$ false-positives increases slightly.

The obvious solution to this problem is spiking *both* $M_\alpha$ and $M_\beta$ in the driver. The effect of such a spiking is depicted in Figure 5d. It is seen that the value of $\Psi_\alpha$ decreases when increasing amounts of $M_\alpha$ and $M_\beta$ are spiked. The decrease is less than the decrease seen when only $M_\alpha$ is spiked to the same extent. This will also be true for $\Psi_\beta$, and the false-positives arising from both $M_\alpha$ and $M_\beta$ will be reduced by this spiking strategy. Although spiking both sequences clearly achieves a beneficial effect by decreasing the number of false-positives with increased spiking levels, the effect is attenuated compared to that observed in Figure 5b and any increase in concentration of spike must be more than 1000-fold to achieve a reduction in the number of false-positives to a level comparable to that in the absence of any non-specific interactions.

### Discussion

The probability that a differentially expressed species *s* will be correctly identified as such by suppression subtractive hybridization depends on $f_s$, the fraction of the $M_i^A M_i^B$ duplexes corresponding to this species to all such duplexes present at the end of the second hybridization. We have derived an analytical expression to calculate this concentration. The concentration, and hence the probability of identification, depends on two categories of factors: *system factors*, such as abundance, degree of differential expression ($\kappa_s$) of the gene, the concentration and percentage similarity of non-specifically interacting sequences, and abundance and number of genes that are not differentially expressed in the two samples; and *reaction conditions*, such as the driver excess ($E_1$ and $E_2$) used in each hybridization, and the hybridization times $t_{hyb1}$ and $t_{hyb2}$.

We have used previously reported results on the number of differentially expressed species to estimate the concentration of DNA that will lead to false-positives. The results for a different number of genes with unchanged expression level can be easily computed. The nature of the results presented here does not change appreciably if a different (lower) number of species is assumed to be present in equal concentrations in the tester and driver (results not shown). We have used reported values of the hybridization rate constants to carry out the simulations. Simulations using values that differ by one order of magnitude from this value show that the results are qualitatively identical to those presented here.

We used the model to predict the effect of changes in the reaction conditions on the probability of identification of genes from different abundance classes that are differentially expressed at various levels. The model predicts that, for the process conditions typically used, the SSH method will lead to the identification of some differentially expressed genes, but will also yield a high number of false-positives. A number of studies have reported false-positive results as high as 90% (Nemeth *et al.*, 2000a, 2000b).

The probability of identification is also a function of transcript length. Each cDNA is digested with *RsaI*, which results in multiple fragments of the same gene being present. As these fragments undergo independent hybridization reactions, the probability of identification of a particular gene is approximately proportional to the number of fragments, and hence to transcript length. Conversely, a long gene that is not differentially expressed will have a higher contribution to the number of false-positives than is estimated by the model. A number of studies have reported that all the isolated sequences at the end of the SSH process are unique (Glienke *et al.*, 2000; Grillari *et al.*, 2000; Sandhu *et al.*, 2000; Shen and Gudas, 2000; Wang *et al.*, 1999). This study suggests that if there is a high efficiency of subtraction, redundant clones will be obtained, either in the form of multiple colonies having the same insert, or colonies with different fragments of the same gene.

From the analytical solution to the coupled set of differential equations, it is possible to calculate the normalized partial derivative of the relative concentration of the $M_i^A M_i^B$ duplex with respect to process parameters. To increase the probability of isolating a differentially expressed gene *i*, the process parameter values should be optimized to increase $\Psi_i$. The value of the partial derivative provides a

Copyright © 2002 John Wiley & Sons, Ltd. 

quantitative estimate of the effect of a proposed change in one process parameter. It should be noted that the value of the partial derivative, by definition, is true only in a small interval near the values of the parameters at which it is estimated. Using the analytical expression presented here, such a calculation is easy to implement for values different from those presented here.

In addition to determining the extent of the false-positives that result from the SSH process, the nature of the false-positives can be explored using our model. In the absence of non-specific interactions, $\Psi_i$ for abundant species with $\kappa_i = 1$ is less than the corresponding concentration for rare species. The basis of this counterintuitive observation lies in the slower kinetics of duplex formation for residual rare single-stranded species with $\kappa_i = 1$ compared to the corresponding abundant sequences and does appear in the transient concentration profiles (data not shown).

### Effect of non-specific hybridization

In the cDNA mixture used for the SSH process, sequences corresponding to proteins with conserved motifs exhibit partial complementarity to each other. Chimeric duplexes formed between strands having partial sequence homology have been observed at the end of the SSH process, in some cases at levels as high as 2% of all duplexes (Zhang *et al.*, 2000). Such non-specifically interacting sequences include those that are differentially expressed, and those that are present at equal concentrations in the tester and driver. In our simulation we specifically address the issue of false-positives caused by the latter type of sequences. Simulation results predict that if there exist two abundant sequences with at least 94% homology, the false-positive pool will be dominated by these sequences. Experimental results from an investigation (Korke *et al.*, unpublished results) of the expressed species in a hybridoma cell culture reveal that the predominant false-positive obtained during a SSH belonged to a class of molecules called intracisternal A particles, reiterated murine retrovirus-like elements (Dupressoir *et al.*, 1999) with high intrasequence similarity (Leib-Mosch *et al.*, 1992; Rynditch *et al.*, 1998). Microarray and Northern blot analysis reveals that these sequences are equally expressed in the hybridoma samples under consideration in the SSH study (Korke *et al.*, unpublished results). The observation that a large fraction of the false-positive pool consists of IAP sequences supports the simulation results presented here.

The addition of specific sequences to the driver in order to reduce the concentration of a particular sequence in the final products has been reported in other subtractive hybridization approaches such as cDNA RDA (Hubank and Schatz, 1994). There are several possibilities in the choice of the sequence that is to be added or 'spiked' to the driver. Either, or both partially complementary sequences, or a consensus sequence, or a concatenated sequence may be used. We used the mathematical model to simulate the efficacy of these approaches in reducing the concentration of the target sequence in the final product mix. The effect of spiking any one of the two sequences has a detrimental effect on false-positives resulting from the sequence that is not spiked. However, the concentration of duplexes corresponding to the spiked sequence decreases appreciably. Spiking a consensus sequence is akin to spiking an interacting sequence, and hence the effect on both sequences will be detrimental. Spiking a sequence that is a concatenation of the two sequences will have an effect that depends on the stability of the duplex containing a large dangling end that will be formed. If this duplex is as stable as a perfectly complementary duplex, the effect will be beneficial. Otherwise, as is thermodynamically more likely, if the duplex thus formed has a significant melting rate, the effect will be analogous to adding a partially complementary sequence. The best result is obtained when both sequences are spiked. Higher spiking levels ($>$1000-fold) have to be used, but a reduction in the levels of both sequences to levels representative of false-positives in the absence of non-specific interactions can be achieved.

Simulation of non-specific interactions has been carried out assuming a homology of 94% between the two sequences. Simulations were also carried out assuming a homology of 77%, for which rate data was available. The rate of the forward reaction is one-hundredth that of perfectly complementary sequences, and the melting rate is high. The false-positive rate obtained is the same as in the case of ideal hybridization (results not shown). Thus, non-specific hybridization between sequences with partial sequence homology of less than 80% does not affect the SSH performance.

Copyright © 2002 John Wiley & Sons, Ltd. 

In an actual experiment, there might be more than two sequences with a high homology. For a particular sequence $\alpha$, the key step that determines $\Psi_\alpha$ is the formation and slow dissociation of heteroduplexes formed from $M_\alpha^A$(or $M_\alpha^B$) and a driver strand of the interacting sequence $M_\beta^D$, as is observed from an examination of the temporal kinetics during the hybridization processes (results not shown). The exact composition of the interacting sequence is not important, and therefore we contend that the simulations for a one-interactor case may be taken as representative of a situation where multiple non-specifically interacting sequences are present. The effect of non-specific interactions on species that are differentially expressed and/or under different process conditions can be easily simulated using the model presented here. It is seen that for extreme process conditions ($t_{hyb} = 48$ h, $E = 300$), the presence of an abundant non-differentially expressed sequence with 94% sequence complementarity increases $\Psi_\alpha$ values for differentially expressed rare and intermediate abundance species. However, the concentration of false-positives also increases (results not shown).

It is seen that there is a differential effect, both qualitative and quantitative, of changing process conditions on the probability of isolating differentially expressed transcripts depending on their abundance, degree of differential expression and the presence of non-specific hybridization. For some transcripts, $\Psi_i$ increases with a positive change in the process parameter and for some the relative concentration decreases. Thus, the model aids in probing the effect of a proposed change on the transcript class of interest. This mathematical model will serve as a tool to carry out virtual SSH experiments to determine the best conditions for the particular sample under consideration. The framework presented here can also be used for the analysis of the efficiency of other procedures for the isolation of differentially expressed genes.

### Acknowledgements

This work was supported in part by grants from the National Science Foundation (BES-97272), National Aeronautics and Space Administration (#NAG8-134), and the Academic Health Center, University of Minnesota. We wish to thank the Minnesota Supercomputing Institute for use of computing resources.

### References

Alizadeh AA, Staudt LM. 2000. Genomic-scale gene expression profiling of normal and malignant immune cells. *Curr Opin Immunol* **12**: 219–225.

Anderson MLM, Young BD. 1985. Quantitative filter hybridization. In *Nucleic Acid Hybridisation: a Practical Approach*, BD Hames, SJ Higgins (eds). IRL Press: Oxford and Washington, DC; 73–111.

Bittner M, Meltzer P, Chen Y. *et al.* 2000. Molecular classification of cutaneous malignant melanoma by gene expression profiling. *Nature* **406**: 536–540.

Brenner S, Johnson M, Bridgham J. *et al.* 2000. Gene expression analysis by massively parallel signature sequencing (MPSS) on microbead arrays. *Nature Biotechnol* **18**: 630–634.

Brown PO, Botstein D. 1999. Exploring the new world of the genome with DNA microarrays. *Nature Genet* **21**: 33–37.

Cho TJ, Park SS. 1998. A simulation of subtractive hybridization. *Nucleic Acids Res* **26**: 1440–1448.

Clontech Manual. 1999. *Clontech PCR-Select™ cDNA Subtraction Kit User Manual*. Clontech Laboratories: Palo Alto, CA.

Craig ME, Crothers DM, Doty P. 1971. Relaxation kinetics of dimer formation by self-complementary oligonucleotides. *J Mol Biol* **62**: 383–401.

DeRisi JL, Iyer VR, Brown PO. 1997. Exploring the metabolic and genetic control of gene expression on a genomic scale. *Science* **28**: 681–685.

Diatchenko L, Lau YF, Campbell AP. *et al.* 1996. Suppression subtractive hybridization: a method for generating differentially regulated or tissue-specific cDNA probes and libraries. *Proc Natl Acad Sci USA* **93**: 6025–6030.

Dupressoir A, Barbot W, Loireau MP, Heidmann T. 1999. Characterization of a mammalian gene related to the yeast ccr4 general transcription factor and revealed by transposon insertion. *J Biol Chem* **274**: 31068–31075.

Ermolaeva OD, Lukyanov SA, Sverdlov ED. 1996. The mathematical model of subtractive hybridization and its practical application. *Proc Int Conf Intell Syst Mol Biol* **4**: 52–58.

Ermolaeva OD, Wagner MC. 1995. SUBTRACT: a computer program for modeling the process of subtractive hybridization. *Comput Appl Biosci* **11**: 457–462.

Evans JD, Wheeler DE. 1999. Differential gene expression between developing queens and workers in the honey bee. *Apis mellifera. Proc Natl Acad Sci USA* **96**: 5575–5580.

Glienke J, Schmitt AO, Pilarsky C. *et al.* 2000. Differential gene expression by endothelial cells in distinct angiogenic states. *Eur J Biochem* **267**: 2820–2830.

Gotoh M, Hasegawa Y, Shinohara Y, Shimizu M, Tosu M. 1995. A new approach to determine the effect of mismatches on kinetic parameters in DNA hybridization using an optical biosensor. *DNA Res* **2**: 285–293.

Grillari J, Hohenwarter O, Grabherr RM, Katinger H. 2000. Subtractive hybridization of mRNA from early passage and senescent endothelial cells. *Exp Gerontol* **35**: 187–197.

Groenink M, Aad CJ. 1996. Isolation of delayed early genes associated with liver regeneration using the Clontech PCR-Select™ subtraction technique. *CLONTECHniques* **XI**: 7–8.

Gurskaya NG, Diatchenko L, Chenchik A. *et al.* 1996. Equalizing cDNA subtraction based on selective suppression of polymerase chain reaction: cloning of Jurkat cell transcripts induced by

Copyright © 2002 John Wiley & Sons, Ltd. 

phytohemagglutinin and phorbol 12-myristate 13-acetate. *Anal Biochem* **240**: 90–97.
Hastie ND, Bishop JO. 1976. The expression of three abundance classes of messenger RNA in mouse tissues. *Cell* **9**: 761–774.
Hill AA, Hunter CP, Tsung BT, Tucker-Kellogg G, Brown EL. 2000. Genomic analysis of gene expression in *C. elegans*. *Science* **290**: 809–812.
Hipfel R, Schittek B, Bodingbauer Y, Garbe C. 2000. Specifically regulated genes in malignant melanoma tissues identified by subtractive hybridization. *Br J Cancer* **82**: 1149–1157.
Hubank M, Schatz DG. 1994. Identifying differences in mRNA expression by representational difference analysis of cDNA. *Nucleic Acids Res* **22**: 5640–5648.
Ko MS. 1990. An 'equalized cDNA library' by the reassociation of short double-stranded cDNAs. *Nucleic Acids Res* **18**: 5705–5711.
Lee CK, Klopp RG, Weindruch R, Prolla TA. 1999. Gene expression profile of ageing and its retardation by caloric restriction. *Science* **285**: 1390–1393.
Leib-Mosch C, Bachmann M, Brack-Werner R. *et al.* 1992. Expression and biological significance of human endogenous retroviral sequences. *Leukemia* **6**: 72S–75S.
Liang P, Pardee AB. 1992. Differential display of eukaryotic messenger RNA by means of the polymerase chain reaction. *Science* **257**: 967–971.
Lipshutz RJ, Fodor SP, Gingeras TR, Lockhart DJ. 1999. High density synthetic oligonucleotide arrays. *Nature Genet* **21**: 20–24.
Martin KJ, Pardee AB. 2000. Identifying expressed genes. *Proc Natl Acad Sci USA* **97**: 3789–3791.
Milner JJ, Cecchini E, Dominy PJ. 1995. A kinetic model for subtractive hybridization. *Nucleic Acids Res* **23**: 176–187.
Nemeth E, Millar LK, Bryant-Greenwood G. 2000a. Fetal membrane distention — II. Differentially expressed genes regulated by acute distention *in vitro*. *Am J Obstet Gynecol* **182**: 60–67.
Nemeth E, Tashima LS, Yu ZX, Bryant-Greenwood GD. 2000b. Fetal membrane distention — I. Differentially expressed genes regulated by acute distention in amniotic epithelial (Wish) cells. *Am J Obstet Gynecol* **182**: 50–59.
Oh MK, Liao JC. 2000. DNA microarray detection of metabolic responses to protein overproduction in *Escherichia coli*. *Metab Eng* **2**: 201–209.
Pardinas JR, Combates NJ, Prouty SM, Stenn KS, Parimoo S. 1998. Differential subtraction display: a unified approach for isolation of cDNAs from differentially expressed genes. *Anal Biochem* **257**: 161–168.
Patanjali SR, Parimoo S, Weissman SM. 1991. Construction of a uniform-abundance (normalized) cDNA library. *Proc Natl Acad Sci USA* **88**: 1943–1947.
Rynditch AV, Zoubak S, Tsyba L, Tryapitsina-Guley N, Bernardi G. 1998. The regional integration of retroviral sequences into the mosaic genomes of mammals. *Gene* **222**: 1–16.
Sagerstrom CG, Sun BI, Sive HL. 1997. Subtractive cloning: past, present, and future. *Ann Rev Biochem* **66**: 751–783.
Sandhu H, Dehnen W, Roller M, Abel J, Unfried K. 2000. mRNA expression patterns in different stages of asbestos-induced carcinogenesis in rats. *Carcinogenesis* **21**: 1023–1029.
Sasaki YF, Ayusawa D, Oishi M. 1994. Construction of a normalized cDNA library by introduction of a semi-solid mRNA–cDNA hybridization system. *Nucleic Acids Res* **22**: 987–992.
Shen J, Gudas LJ. 2000. Molecular cloning of a novel retinoic acid-responsive gene, Ha1r-62, which is also upregulated in HoxA-1-overexpressing cells. *Cell Growth Diff* **11**: 11–17.
Simpson KS, Adams MH, Behrendt-Adam CY, Ben Baker C, McDowell KJ. 1999. Identification and initial characterization of calcyclin and phospholipase a(2) in equine conceptuses. *Mol Reprod Dev* **53**: 179–187.
Spiegelman GB, Haber JE, Halvorson HO. 1973. Kinetics of ribonucleic acid-deoxyribonucleic acid membrane filter hybridization. *Biochemistry* **12**: 1234–1242.
Stuart RO, Bush KT, Nigam SK. 2001. Changes in global gene expression patterns during development and maturation of the rat kidney. *Proc Natl Acad Sci USA* **98**: 5649–5654.
Velculescu VE, Zhang L, Vogelstein B, Kinzler KW. 1995. Serial analysis of gene expression. *Science* **270**: 484–487.
Vernier P, Mastrippolito R, Helin C. *et al.* 1996. Radioimager quantification of oligonucleotide hybridization with DNA immobilized on transfer membrane: application to the identification of related sequences. *Anal Biochem* **235**: 11–19.
Wang XK, Li X, Yaish-Ohad S. *et al.* 1999. Molecular cloning and expression of the rat monocyte chemotactic protein-3 gene: a possible role in stroke. *Mol Brain Res* **71**: 304–312.
Wetmur JG. 1976. Hybridization and renaturation kinetics of nucleic acids. *Ann Rev Biophys Bioeng* **5**: 337–361.
Wetmur JG, Davidson N. 1968. Kinetics of renaturation of DNA. *J Mol Biol* **31**: 349–370.
White KP, Rifkin SA, Hurban P, Hogness DS. 1999. Microarray analysis of *Drosophila* development during metamorphosis. *Science* **286**: 2179–2184.
Yang GP, Ross DT, Kuang WW, Brown PO, Weigel RJ. 1999. Combining SSH and cDNA microarrays for rapid identification of differentially expressed genes. *Nucleic Acids Res* **27**: 1517–1523.
Zhang J, Underwood LE, D'Ercole AJ. 2000. Formation of chimeric cDNAs during suppression subtractive hybridization and subsequent polymerase chain reaction. *Anal Biochem* **282**: 259–262.
Zhang L, Zhou W, Velculescu VE. *et al.* 1997. Gene expression profiles in normal and cancer cells. *Science* **276**: 1268–1272.

### Notation

| | |
|---|---|
| *baseline* | Total concentration of tester homoduplex with different adaptors A and B on the two strands corresponding to sequences that are not differentially expressed |
| $D_i$ | Total DNA for sequence i available for ligation |
| $E_1$ | Fold-excess driver added for the first set of hybridizations |
| $E_2$ | Fold-excess driver added for the second hybridization |
| $kb_{i,j}^{p,q}$ | Rate constant of melting of the duplex $M_i^p M_j^q$ ($s^{-1}$) |

Copyright © 2002 John Wiley & Sons, Ltd. 

| | |
|---|---|
| $kf_{i,j}^{p,q}$ | Rate constant of duplex formation when $M_i^p$ and $M_i^q$ form $(M_i^p M_j^q)$ l/(moles s) |
| $M_i^p$ | Concentration of single-stranded cDNA species *i* labelled with adaptor *p* (moles of strands/l) |
| $M_i^p M_j^q$ | Concentration of duplex formed by the hybridization of $M_i^p$ and $M_j^q$. (moles/l) |
| $n_{PCR}$ | Number of PCR cycles |
| $n_T$ | Total number of species |
| $p_s$ | Probability of identification of a particular sequence *s* as being differentially expressed |
| t | Time (s) |
| $t_{hyb1}$ | Time that the first hybridization is allowed to proceed |
| $t_{hyb2}$ | Time that the second hybridization is allowed to proceed |
| $Z_{parameter}$ | Normalized partial derivative of $\Psi$ with respect to the *parameter* |
| $\kappa_i$ | Differential expression ratio (ratio of concentrations of a particular species *i* in tester and driver) |
| $\Psi_i$ | Duplex : baseline ratio $\frac{[M_i^A M_i^B]}{[\text{baseline}]}$ |

### Subscripts

| | |
|---|---|
| i and j | Species i and j, respectively; i, j = 1, ..., $n_T$ |
| $\alpha$ and $\beta$ | Species $\alpha$ and $\beta$ that have partial sequence homology |
| 0,1 | Concentrations at the start of the first hybridization |
| 0,2 | Concentrations at the start of the second hybridization |

### Superscripts

| | |
|---|---|
| A | Tester species with adaptor A |
| B | Tester species with adaptor B |
| D | Species present in driver (no adaptor) |
| * | Sequences with $\kappa_i = 1$ |

### Appendix: ideal hybridization model

The balance equations for species *i* during hybridization 1A are:

$$\frac{d[M_i^A]}{dt} = -k_f[M_i^A]^2 - k_f[M_i^A][M_i^D] \quad (A.1)$$

$$\frac{d[M_i^D]}{dt} = -k_f[M_i^D]^2 - k_f[M_i^A][M_i^D] \quad (A.2)$$

$$\frac{d([M_i^A M_i^A]}{dt} = \frac{k_f[M_i^A]^2}{2} \quad (A.3)$$

$$\frac{d([M_i^D M_i^D])}{dt} = \frac{k_f[M_i^D]^2}{2} \quad (A.4)$$

$$\frac{d([M_i^A M_i^D])}{dt} = k_f[M_i^A][M_i^D] \quad (A.5)$$

At the start of this hybridization (t = 0), all species are in the form of single strands. The initial conditions are listed in Table 1. A key factor affecting the outcome of SSH is the extent of differential expression between the tester and driver samples. We define the differential expression ratio $\kappa_i$ as the ratio of the mRNA concentration of species *i* in the tester to that in the driver. The excess ratio E is defined as the ratio of the concentration of total cDNA present in the driver to the total cDNA concentration in the tester.

These equations can be solved for $[M_i^A]$ and $[M_i^D]$ to yield the concentrations of single-stranded tester (A.6), driver (A.7), and duplex species (A.8–A.10).

$$[M_i^A] = \frac{[M_i^A]_{0,1}}{([M_i^A]_{0,1} + [M_i^D]_{0,1})k_f\, t + 1};$$

$$[M_i^D] = \frac{[M_i^D]_{0,1}}{([M_i^A]_{0,1} + [M_i^D]_{0,1})k_f\, t + 1} \quad (A.6,7)$$

$$[M_i^A M_i^A] = \frac{\{[M_i^A]_{0,1}\}^2 k_f\, t}{([M_i^A]_{0,1} + [M_i^D]_{0,1})k_f\, t + 1} \quad (A.8)$$

$$[M_i^A M_i^D] = \frac{[M_i^A]_{0,1}[M_i^D]_{0,1} k_f\, t}{([M_i^A]_{0,1} + [M_i^D]_{0,1})k_f\, t + 1};$$

$$[M_i^D M_i^D] = \frac{\{[M_i^D]_{0,1}\}^2 k_f\, t}{([M_i^A]_{0,1} + [M_i^D]_{0,1})k_f\, t + 1} \quad (A.9,10)$$

Setting $t = t_{hyb1}$ in Equations A.6–A.10, the concentrations at the end of the first hybridization can be obtained. In hybridizations 1A and 1B, the initial concentration of corresponding species is identical, since the tester is divided into two equal parts. As the reaction conditions for both hybridizations are identical, hybridization 1B can be represented by the same set of equations (Equations A.1–A.10) with the superscript B replacing A.

Copyright © 2002 John Wiley & Sons, Ltd. 

The balance equations for single stranded species, homoduplexes with identical adaptors, and homoduplexes with different adaptors during the second hybridization are given by Equations A.11, A.12 and A.13, respectively:

$$\frac{d[M_i^p]}{dt} = -k_f[M_i^A][M_i^p] - k_f[M_i^D][M_i^p] - k_f[M_i^B][M_i^p];\ p = A,B,D \quad (A.11)$$

$$\frac{d([M_i^p M_i^p])}{dt} = \frac{k_f[M_i^p]^2}{2};\ p = A,B,D \quad (A.12)$$

$$\frac{d[M_i^p M_i^q]}{dt} = k_f[M_i^p][M_i^q];\ p,\ q = A,\ B,\ D;\ p \neq q \quad (A.13)$$

At the start of the second hybridization, equal volumes of the products of hybridizations 1A and 1B (without melting), and fresh melted single-stranded driver are mixed. The concentration is thus reduced by a factor of three. Substituting $[M_i^B] = [M_i^A]$ in Equation A.11, we can write the balance equation for species $M_i^A$ and $M_i^D$ as:

$$\frac{d[M_i^A]}{dt} = -2k_f[M_i^A]^2 - k_f[M_i^A][M_i^D];$$

$$\frac{d[M_i^D]}{dt} = -k_f[M_i^D]^2 - 2\ k_f[M_i^A][M_i^D] \quad (A.14;\ 15)$$

Solving for $M_i^A$ and $M_i^D$, with initial conditions listed in Table 1, we obtain:

$$[M_i^A] = \frac{[M_i^A]_{0,2}}{(2[M_i^A]_{0,2} + [M_i^D]_{0,2})k_f\ t + 1};$$

$$[M_i^D] = \frac{[M_i^D]_{0,2}}{(2[M_i^A]_{0,2} + [M_i^D]_{0,2})k_f\ t + 1} \quad (A.16;\ 17)$$

The rate expression for the concentration of $M_i^A M_i^B$ is given by Equation A.13. As $[M_i^B] = [M_i^A]$, we can write:

$$\frac{d[M_i^A M_i^B]}{dt} = k_f[M_i^A][M_i^B] = k_f[M_i^A][M_i^A] \quad (A.18)$$

Substituting Equation A.16 in Equation A.18 and integrating using the initial conditions given in Table 1, we get:

$$[M_i^A M_i^B] = \frac{\{[M_i^A]_{0,2}\}^2 k_f\ t}{(2[M_i^A]_{0,2} + [M_i^D]_{0,2})k_f\ t + 1} \quad (A.19)$$

Substituting for $[M_i^A]_{0,2}$ and $[M_i^D]_{0,2}$ in Equation (A.19) an analytical expression for $[M_i^A M_i^B]$ at the end of the second hybridization ($t = t_{hyb2}$) in terms of the initial concentration, $\kappa_i$, $E_1$, $E_2$, $t_{hyb1}$ and $t_{hyb2}$ can be obtained (Equation 3).

Copyright © 2002 John Wiley & Sons, Ltd.
